# Mimicking the Organic and Inorganic Composition of Anabolic Bone Enhances Human Mesenchymal Stem Cell Osteoinduction and Scaffold Mechanical Properties

**DOI:** 10.3389/fbioe.2020.00753

**Published:** 2020-07-03

**Authors:** Eli Mondragón, Mitzy Cowdin, Francesca Taraballi, Silvia Minardi, Ennio Tasciotti, Carl A. Gregory, Roland Kaunas

**Affiliations:** ^1^Department of Biomedical Engineering, Texas A&M University, College Station, TX, United States; ^2^Center for Musculoskeletal Regeneration, Houston Methodist, Houston, TX, United States; ^3^Department of Molecular and Cellular Medicine, Texas A&M Health Science Center, College Station, TX, United States

**Keywords:** mesenchymal stem cells, extracellular matrix, drug-delivery, bioreactor, bone, biomimetic

## Abstract

Engineered bone graft designs have been largely inspired by adult bone despite functionally significant differences from the composition of anabolic bone in both the mineralized and non-mineralized fractions. Specifically, anabolic bone contains hydroxyapatite with ionic substitutions that facilitate bone turnover and relatively rare collagens type VI and XII that are important for normal bone development. In this work, human mesenchymal stem cells (hMSCs) were cultured in lyophilized collagen type I scaffolds mineralized with hydroxyapatite containing Mg^2+^ substitutions, then induced to deposit an extracellular matrix (ECM) containing collagens VI and XII by exposure to GW9662, a PPARγ inhibitor. Delivery of GW9662 was accomplished through either Supplemented Media or via composite microspheres embedded in the scaffolds for localized delivery. Furthermore, hMSCs and scaffolds were cultured in both static and perfuse conditions to investigate the interaction between GW9662 treatment and perfusion and their effects on ECM deposition trends. Perfusion culture enhanced cell infiltration into the scaffold, deposition of collagen VI and XII, as well as osteogenic differentiation, as determined by gene expression of osteopontin, BMP2, and ALP. Furthermore, scaffold mineral density and compressive modulus were increased in response to both GW9662 treatment and perfusion after 3 weeks of culture. Local delivery of GW9662 with drug-eluting microspheres had comparable effects to systemic delivery in the perfusate. Together, these results demonstrate a strategy to create a scaffold mimicking both organic and inorganic characteristics of anabolic bone and its potential as a bone graft.

## Introduction

Bone repair recapitulates several ontological events that occur during both skeletal development and post-natal bone growth (Dirckx et al., 2013; Einhorn and Gerstenfeld, 2015). The anabolic environment is characterized by the recruitment of progenitor cells and the deposition of *de novo* tissue that undergoes extensive remodeling (Gerstenfeld et al., 2003; Little et al., 2007; Dirckx et al., 2013; Einhorn and Gerstenfeld, 2015). While collagen type I is a major component of the organic phase of homeostatic bone, there are a number of other collagen types that are found in relative abundance in embryonic and regenerating bone (Wälchli et al., 1994; Marvulli and Bressan, 1996; Yamazaki et al., 1997). Collagen types VI (Coll VI) and XII (Coll XII) are upregulated in developing bone, where they play crucial roles in regulating bone growth (Wälchli et al., 1994; Kohara et al., 2015, 2016). Furthermore, approximately 70% of the dry weight of mature bone is composed of impure, low crystallinity hydroxyapatite (HA). Although HA is typically depicted stoichiometrically as Ca_10_(PO_4_)_6_(OH)_2_, cationic and anionic substitutions in the crystalline structure are quite common (Landi et al., 2008). Specifically, Mg^2+^ is abundant in bone during the initial phases of osteogenesis and disappears in mature bone (Landi et al., 2008). Despite the known differences in composition between anabolic and homeostatic bone, engineered bone grafts have not previously been designed to resemble both the organic and inorganic composition of regenerating bone.

Cationic (e.g., Zn^2+^, Mn^2+^, Mg^2+^) and anionic (e.g., CO_3_^2–^, Fl^–^, Cl^–^, SiO_4_^4–^) substitutions in the lattice structure of bone mineral have motivated the development of a wide range of ion-substituted HA for bone repair (Ratnayake et al., 2017). Among these ions, Mg is unique in that it is relatively abundant in bone during development and repair. Mg-containing biomaterials have shown promise when applied to bone repair. Mg-based ceramics enhanced the osteogenic (Su, 2018) and resorption properties of scaffolds (He et al., 2014). In fracture fixation, Mg stimulated new bone formation when incorporated into degradable screws and plates (Chaya et al., 2015). However, tailoring the mineral content of scaffolds can only recapitulate the inorganic fraction of the anabolic niche.

ECM deposited by rat mesenchymal stem cells (MSCs) onto titanium mesh (Datta et al., 2005) or human MSCs (hMSCs) onto tissue culture plastic (Decaris et al., 2012) promote osteogenic differentiation of freshly seeded MSCs. We have developed a protocol that uses GW9662, a PPARγ inhibitor, to induce osteogenic differentiation of hMSCs, during which time the cells generate an ECM rich in Coll VI and XII (Zeitouni et al., 2012; Clough et al., 2015). Depositing this ECM onto gelatin foam followed by decellularization results in a graft that accelerates bone healing in mice (Clough et al., 2015; Sears et al., 2020). Translation of this technology to the clinic would be facilitated by a strategy that allows the ECM to be generated after implantation through sustained local delivery of GW9662 within the scaffold.

For the purpose of this study, we utilized a biologically inspired osteoinductive scaffold—a macroporous collagen scaffold coated with Mg-doped HA (Coll/MgHA)—as previously reported (Minardi et al., 2015). To mimic the organic fraction of nascent bone, seeded hMSCs were stimulated with GW9662 to deposit Coll VI and Coll XII on the scaffolds. We hypothesized that incorporating a drug delivery system capable of controlled release of GW9662 into the Coll/MgHA scaffold would stimulate seeded hMSCs to deposit similar levels of the anabolic bone ECM as that of induced with GW9662 added directly to the media. To achieve prolonged GW9662 release, a drug-eluting platform consisting of porous silica particles (pSi) encapsulated in poly(lactide-co-glycolic acid) (PLGA) microspheres was used (Fan et al., 2012; Minardi et al., 2014; Pandolfi et al., 2016). Furthermore, a bioreactor was used to perfuse the scaffold to mimic the gas and nutrient transport environment observed during neovascularization of callus tissue (Dirckx et al., 2013).

## Materials and Methods

### Preparation of GW9662-Loaded PLGA/pSi Microspheres

The drug-eluting composite microspheres consist of porous silica (pSi) suspended in poly(lactic-co-glycolic) (PLGA) microspheres using methods previously described (Tsao et al., 2018). Unless stated otherwise, the reagents were purchased from Sigma-Aldrich (St. Louis, MO, United States). Briefly, 300 μl of tetraethyl orthosilicate was added dropwise to a mixture of 0.36% (w/v) tannic acid, 66% (v/v) ethanol, and 33% (v/v) ammonium hydroxide. The mixture was stirred continuously for 3 h before the pSi were washed and centrifuged three times in a 1:1 solution of ethanol:water. The pSi particles were then lyophilized and stored at −20°C until needed. To load the pSi with GW9662, 5 mg of lyophilized pSi were incubated in 1 mL of GW9662 (300 μg/mL) dissolved in DMSO with constant stirring for 20 min at 37°C. The GW9662-loaded pSi were washed and lyophilized before proceeding to encapsulation in PLGA microspheres. Briefly, GW9662-loaded pSi were added to a 5% (w/v) PLGA (50:50) (Lactel Absorbable Polymers, Pelham, AL, United States) solution dissolved in dichloromethane (DCM). The PLGA/pSi/DCM mixture was then added dropwise to a 2.5% poly(vinyl alcohol) (PVA) aqueous solution and stirred continuously for 6 h. The resulting microspheres were then centrifuged and washed several times with DI water before they were lyophilized and stored at −20°C until needed.

### Particle Characterization and Evaluation of GW9662 *in vitro* Release

To verify the presence of pSi in PLGA microspheres, fluorescein isothiocyanate (FITC)-labeled pSi were encapsulated in PLGA microspheres and imaged by confocal microscopy (Nikon D Eclipse C1, Nikon Corporation, Japan). Liquid chromatography-mass spectrometry was used to measure the release of GW9662 from the PLGA-pSi microspheres suspended in an aqueous buffer over the course of 21 days.

### Scaffold Fabrication

Porous Coll/MgHA scaffolds with and without PLGA-pSi microspheres were fabricated as described previously with minor modifications (Minardi et al., 2015) (Supplementary Figure 1A). Type I bovine collagen (Nitta Casings, Bridgewater, NJ, United States) was chosen as the organic matrix upon which Mg-doped HA nanocrystals would nucleate during collagen fibrils self-assembly. Briefly, 200 mg of collagen was dissolved in 20 mL of acetic acid buffered at a pH of 3. To the collagen solution, 190 μL of 85% H_3_PO_4_ (Sigma-Aldrich) and 20 mL of DI water were added. Collagen fibrils containing HA and MgHA were reconstituted by adding the acidic collagen solution dropwise to a basic suspension consisting of 0.35 g of Ca(OH)_2_ and 0.166 g of MgCl_2_⋅6H_2_O (Thermo Fisher, Waltham, MA, United States) in 20 mL of water. The ratio of Ca(OH)_2_ and MgCl_2_⋅6H_2_O in the basic suspension was set to 15% as a higher ratio was needed in the precursor solution to achieve the 5–6% molar substitution of Mg^2+^ in the final lattice structure. The mixture was aged for 24 h at 37°C followed by wet crosslinking with 1,4 butanediol diglycidyl ether (BDDGE) (2.5 mM) (Sigma-Aldrich) at 4°C for 48 h at a pH of 8. The mixture was then centrifuged and washed several times with DI water to remove crosslinking solution, leaving behind a slurry-like mixture. In some cases, drug-eluting microspheres were added to achieve approximately 0.52 μg GW9662/scaffold. To fabricate a porous structure, the slurry was aliquoted into 96-well plates (70 μL/well) and lyophilized after undergoing controlled freezing (Minardi et al., 2015). The resulting Coll/MgHA scaffolds, referred to either MgHA or MgHA/pSi for brevity, were then stored at −20°C until needed.

### Structural and Compositional Characterization of Scaffolds

Fourier-transformed Infrared (FTIR) was performed using an Alpha-Platinum Bruker Spectrometer (Billerica, MA, United States). Prior to acquiring their FTIR spectra, the porous scaffolds were flattened into thin disks using a mortar and pestle and were then kept at 100°C in a heating block for an hour to remove residual water. Thermogravimetric analysis (TGA) was performed on a Q50 (TA Instrument, New Castle, DE, United States) by heating scaffolds (<10 mg) from 25 to 1000°C at a rate of 10°C/min. The molar ratio of Mg^2+^ to Ca^2+^ present in the mineral phase of the scaffolds was determined through inductively coupled plasma-mass spectrometry (ICP-MS) (PerkinElmer NexION 300D, Waltham, MA, United States), using a multi-element reference standard of Ca^2+^ and Mg^2+^ (ICP CAL-STD EARTH ALKALI, Inorganic Ventures, Christiansburg, VA, United States) in 1% nitric acid. For ICP-MS, scaffolds were digested overnight in 1% nitric acid. The supernatant was collected after a brief centrifugation, filtered using a 0.22 μm syringe filter, and diluted in 1:1200 in 1% nitric acid to ensure ion concentration were within standard curves. Finally, the morphology and distribution of surface pore size of the scaffolds were assessed by scanning electron microscopy (SEM) on a JCM 5000 (JEOL, Peabody, MA, United States) and analyzed using ImageJ (NIH Image). Lyophilized scaffolds stored in −20°C were kept overnight in a desiccator and were then gold sputter coated (20 mA, 60 s) to remove accumulated water prior to SEM imaging. Approximately 146 and 119 pores were analyzed for the MgHA and MgHA/pSi scaffolds, respectively.

### Expansion and Seeding of hMSCs

Bone marrow-derived hMSCs were acquired from the adult stem cell distribution center at Texas A&M Health Science Center Institute for Regenerative Medicine in accordance with institutionally approved protocols. Bone marrow-derived hMSCs were expanded in complete culture media (CCM) consisting of alpha minimal essential medium (α-MEM, Invitrogen, Carlsbad, CA, United States), 20% (v/v) FBS (Atlanta Biologicals, Flowery Branch, GA, United States), 2 mM L-glutamine (Invitrogen), and 100 U/ml penicillin plus 100 μg/ml streptomycin (HyClone^TM^ Marlborough, MA, United States) at a seeding density of 500 cells/cm^2^. Culture media was changed every 2–3 days until cells reached 70–80% confluency. Expanded cells were then recovered by brief trypsinization (Corning, Corning, NY, United States) and seeded at the top of each scaffold (200,000 cells/scaffold) using a 20 μL aliquot. After incubating for 10 min, scaffolds were flipped 180° and an additional 5 μL of CCM was added to achieve a more uniform distribution of cells. Scaffolds were then incubated for an additional 10 min before CCM was added to well plates, keeping track of the area in which cells were initially seeded. The cells were incubated overnight before scaffolds were switched to different culture media formulations or placed in the perfusion bioreactor. The other culture media formulations included: (1) osteogenic basal media (OBM) consisting of CCM supplemented with 50 μg/mL of ascorbic acid and 5 mM β-glycerol phosphate, (2) OBM supplemented with 10 μM GW9662 dissolved in DMSO as a positive control, and (3) OBM supplemented with an equal volume of DMSO as a vehicle control. A table depicting the scaffold/media formulation used for osteogenic assays can be found in Table 1.

**TABLE 1:** Scaffold composition and cultured media used for osteogenic assays.

**Scaffold**	**Media formulation**	**Denoted as**
Collagen/MgHA	OBM + DMSO (vehicle control)	DMSO
Collagen/MgHA	OBM + 10 μM GW9662 (positive control)	GW-media
Collagen/MgHA + PLGA/pSi	OBM (controlled GW9662 delivery)	GW-pSi

*All possible permutations of scaffold/media formulations were cultured in both static and perfused conditions.*

### Culture in Static and Perfused Conditions

Scaffolds were placed in the wells of the bioreactor (Supplementary Figures 1B,C), positioning them so that surface in which cells were initially seeded faced down toward the channels. A silicone rubber gasket with a diameter similar to the scaffolds was placed on top to block perfusate from flowing around, instead of through, the scaffolds. Once placed in the perfusion bioreactor, a multichannel peristaltic pump (Cole-Palmer, Vernon Hills, IL, United States) was used to adjust the culture media flow rate to achieve a superficial velocity through the scaffolds of approximately 116 μm/s, resulting in an estimated shear stress of about 7 mPa. Shear stress experienced by the cells was estimated using a previously described equation (Grayson et al., 2008):

$$\tau_{w}=\frac{8\,\mu_{m}\,u}{d_{p}}$$

where τ_w_ is the fluid wall stress within the pores of the scaffold, μ_m_ is the viscosity (∼0.77 cPa) of the culture media, *u* is the interstitial velocity, and *d*_p_ is the average pore diameter observed in the scaffolds by SEM imaging. Scaffolds were perfused up to 21 days, with media changes occurring every 2–3 days.

### Cell Migration on Scaffolds

Seeding efficiency was approximated by recovering hMSCs attached to the well plates rather than the scaffolds the day after seeding using standard cell counting procedure with a hemacytometer. To evaluate hMSCs viability and migration into the scaffolds without the influence of soluble factors, live-dead staining—5 μM of Calcein AM and 0.1% Propidium Iodide—was performed after 8 days of static and perfused culture in MgHA/CCM scaffolds. Confocal Z-stack images of the scaffolds were captured with a Nikon D Eclipse C1 upright microscope (Nikon Corporation, Japan) from upstream face of the scaffold to a depth of 200 μm using 10 μm slices. The relative fluorescent intensity units for z-stacked images were evaluated as a function of depth using ImageJ (NIH Image). The depth at which stacked live-dead images decreased to 50% of maximum fluorescence intensity was quantified for both static and perfused samples. Afterward, scaffolds were cut to acquire cross-sectional images.

### Immunostaining of Deposited Matrix Proteins

GW9662-treated hMSCs have been previously shown to deposit a unique ECM rich in collagens that are highly expressed in anabolic bone, specifically Coll VI and Coll XII. To evaluate the efficacy of the PLGA-pSi microspheres, immunostaining for Coll VI and XII was performed on scaffolds after 8 days of culture. Briefly, scaffolds were fixed overnight at 4°C in 4% formaldehyde in PBS and washed three times with PBS. Scaffolds were then blocked with 5% goat serum (MP Biomedical) and 0.3% Triton-X (Sigma Aldrich) for 1 h at room temperature before being incubated overnight at 4°C in blocking buffer supplemented with either rabbit anti-Human Coll VI or Coll XII (1:200, Novus Biological, Littleton, CO, United States). Scaffolds were then washed with PBS and were incubated at room temperature for 2 h in blocking buffer with fluorescein-conjugated goat anti-rabbit (1:500, Millipore, Burlington, MA, United States). Z-stack images were taken as described above. Maximum intensity projections was performed on the EZ-C1 software (Nikon Corporation, Japan).

### Gene Expression via Quantitative RT-PCR (qRT-PCR)

After 8 and 21 days of culture, total RNA was extracted from the scaffolds with RNeasy Mini Kit (Qiagen, Hilden, Germany) supplemented with Trizol (Life Technologies, Carlsbad, CA, United States). After quantifying the purity and concentration of RNA (Infinite M200 Pro, Tecan, Männedorf, Switzerland), isolated RNA with a purity (A260/A280) above 1.9 was used for cDNA synthesis in 21 μL reaction (Superscript III kit, Invitrogen). Approximately 6.5 ng of cDNA was amplified in a 20 μL reaction containing Brilliant III Ultra-Fast SYBR Green QPCR Master Mix with Low ROX (Agilent, Santa Clara, CA, United States) on an Agilent Aria Mx Real-Time PCR System. The primer list and their sequences can be found in the Supplementary Information (Supplementary Table 1). Relative expression was calculated using the ΔΔC_T_ method normalized to human glyceraldehyde-3-phosphate dehydrogenase (GAPDH) levels, using hMSCs cultured for either 8 or 21 days in 2D monolayer on tissue culture plate with CCM as the calibrator control. Relative fold change in gene expression was calculated using 2^–ΔΔCt^ method described by Livak and Schmittgen (2001). Statistical comparisons were performed on GraphPad Prism software (San Diego, CA, United States). Briefly, a Bartlett’s test was performed on the C_t_ values for each gene, for each scaffold/culture condition (Supplementary Table 5) to confirm homogeneity prior to running a two-way ANOVA. A two-way ANOVA was performed on the calculated ΔΔC_T_ values, followed by a Tukey’s multiple comparison test (*n* = 3), whose results can be found in the Supplementary Information (Supplementary Tables 5–17). In addition, the number of hMSCs present in the scaffolds was measured via GAPDH expression after 8 days of static and perfused culture in all scaffold/culture conditions using known cell number standards. RNA was extracted from the cell standards in the presence of MgHA scaffolds to mimic the RNA extraction efficiency from the cultured scaffolds. Statistical testing as described above was performed on the calculated cell number (Supplementary Tables 2–4).

### Microcomputed Tomography (μCT) Analysis of Cultured Scaffolds

After 21 days of static and perfused cultures, scaffolds were washed with PBS and fixed overnight at 4°C with 4% formaldehyde in PBS. After washing with and aspirating excess PBS, samples were allowed to dry overnight in a desiccator. Samples were stored at −80°C until needed. Uncultured scaffolds were used as controls and were prepared as described above. Prior to acquiring μCT scans on a SkyScan 1275 X-Ray Microtomograph (Bruker, Billerica, MA, United States), samples were gently wrapped in parafilm for easier handling during imaging on the SkyScan’s rotating stage. Scaffolds were imaged over 360 degrees with a camera resolution of 18 μm, with images taken every 0.5 degrees using a 28 kV beam and a frame averaging of 3. The cross-sectional images of the scaffolds were reconstructed using NRecon (Micro Photonics) software, keeping the smoothing and beam-hardening compensation settings the same between samples at 0 (Gaussian Kernel) and 41%, respectively. Thresholding was done to better distinguish between parafilm and the scaffolds. Calcium hydroxyapatite phantoms (0.25 and 0.75 g/cm^3^; Bruker) were scanned using the settings described above and analyzed in the Bruker-MicroCT CT-Analyzer (CTan) software to determine their attenuation coefficient values in order to predict the bone mineral density (BMD) of the scaffolds. After confirming equal variances (Supplementary Table 18), a two-way ANOVA followed by a Tukey’s multiple comparison test were performed on the bone mineral density values (*n* = 3). Both the ANOVA table and the results for the multiple comparison test can be found in the Supplementary Information (Supplementary Tables 19, 20). CTan software was also utilized to measure morphological characteristics of the scaffolds, specifically percent porosity and the specific bone surface (BS/BV) (Bouxsein et al., 2010) of the scaffolds. The CTVox software was utilized to reconstruct images of the cultured samples. Identical opacity thresholding was utilized for all reconstructions. Maximum intensity projection (MIP) was enabled to highlight dense regions of mineralization.

### Uniaxial Compression Test

After acquiring μCT scans, scaffolds were gently removed from the parafilm wrapping and stored at −80°C until needed for mechanical testing. The diameter and thickness of the scaffolds were measured with calipers, and underwent a uniaxial compression test to 15% strain at a rate of 5 μm/s on a DMA 850 (TA Instruments). The compressive modulus was calculated through linear fitting of the slope. Statistical comparisons were performed on the compressive modulus using two-way ANOVA followed by a Tukey’s multiple comparison test (*n* = 3). The ANOVA table and its corresponding multiple comparison results can be found in the Supplementary Information (Supplementary Tables 22, 23).

### Immunoblotting

After 8 days of static and perfused culture, scaffolds were washed with warm followed by ice-cold PBS. Proteins were extracted using ice-cold RIPA buffer (50 mM Tris HCl, 150 mM NaCl, 1% Triton-X-100, 0.1% SDS, 0.5% Sodium Deoxylcholate, 1 mM EDTA, 1 mM Sodium Pyrophosphate) supplemented with SIGMAFAST^TM^ protease inhibitors (Sigma-Aldrich) through vortexing followed by centrifugation at 12,000 *g* for 10 min. The supernatant was recovered and stored at −80°C until total protein content was quantified through Pierce BCA Protein Assay kit (Thermo Fisher Scientific). Protein samples (15 μg) were resolved in hand-casted 10% SDS-PAGE gel (Bio-Rad, Hercules, CA, United States) and transferred to a PVDF membrane (Millipore). The membrane was blocked in 5% milk in TBST buffer (20 mM Tris-base, 150 mM NaCl, 0.2% Tween 20) for 1 h before being incubated overnight at 4°C with mouse anti human BMP2 antibody (R&D Systems, Minneapolis, MN, United States) diluted (1:500) in blocking buffer. After three washes in TBST buffer, the membrane was incubated for 1 h with an HRP-conjugated goat anti mouse secondary antibody (Protein Tech, Rosemont, IL, United States). The membrane was developed for 5 min in WesternSure Chemiluminescent Substrate (LI-COR, Lincoln, NE, United States) and imaged on a C-Digit Blot Scanner (LI-COR). After imaging, the membrane was stripped using Restore Stripping Buffer (Thermo Fisher Scientific) following the manufacturer’s instructions. The membrane was then blocked, incubated with mouse-anti-human GAPDH (1:4000, Protein Tech), and imaged as previously described.

## Results

### Morphology and Composition of Scaffolds

**FIGURE 1:**
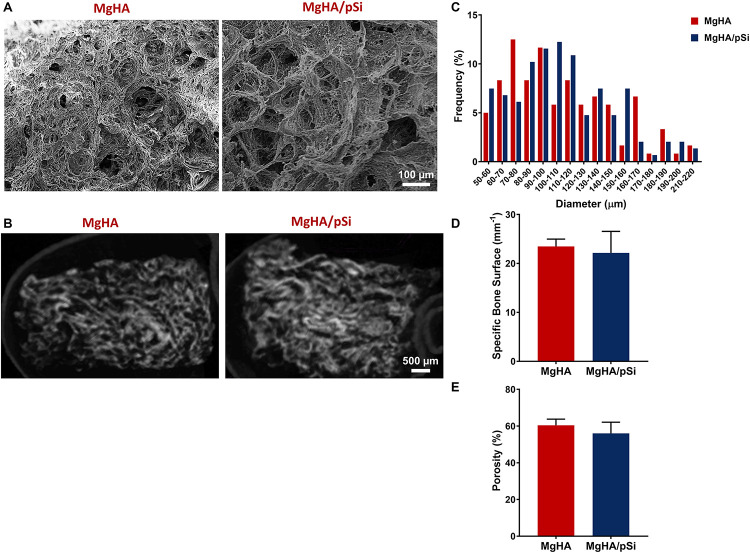
Morphological Characterization of MgHA and MgHA/pSi scaffolds. Representative **(A)** SEM and **(B)** micro-computed tomography (μCT) images of MgHA and MgHA/pSi scaffolds. **(C)** Pore size distribution is shown to be similar between the two scaffolds. **(D)** Specific bone surface ratio and **(E)** percent porosity were unaffected by the addition of drug-eluting microspheres.

The microstructure of MgHA and MgHA/pSi scaffolds were evaluated via SEM and μCT. SEM imaging of the scaffold surfaces revealed an isotropic porous structure (Figure 1A). Cross-sectional μCT scans of the scaffolds internal microstructure indicated interconnected pores (Figure 1B). When quantifying surface pore size, a wide distribution of pore diameter of 50–200 μm was observed (Figure 1C). Analysis of morphological characteristics showed that inclusion of the drug-eluting microspheres had no effect on either specific bone surface ratio (Figure 1D) or porosity (Figure 1E).

**FIGURE 2:**
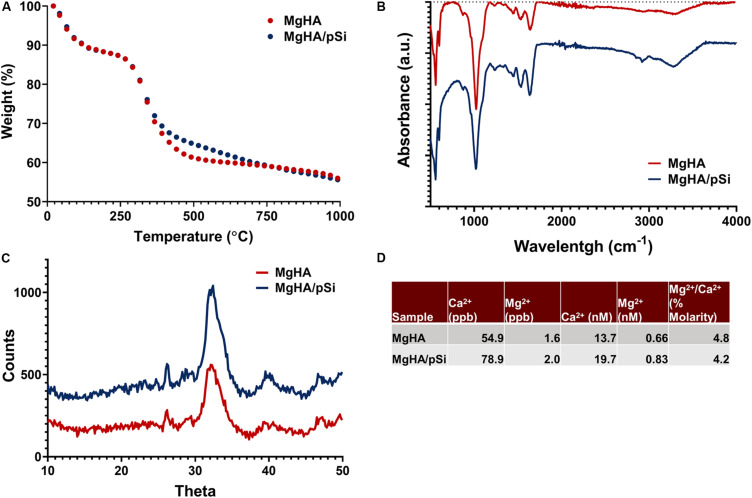
Characterization of the composition of the MgHA and MgHA/pSi scaffolds via TGA **(A)**, FTIR **(B)**, XRD **(C)**, and ICP-MS **(D)**.

TGA analysis was performed to quantify the mineral-to-protein ratio of the scaffolds. The initial loss in mass observed, approximately 10%, was attributed to residual water in the scaffolds. Thus, the collagen matrix and the mineral phase contributed approximately 33 and 66% of the scaffolds dry weight (Figure 2A). Scaffold composition was further characterized using FTIR (Figure 2B). Large bands were observed in 550 cm^–1^ and 1000 cm^–1^, corresponding to phosphates in hydroxyapatite. For the organic component, vibrational bands characteristic to amide groups were observed around 1250 cm^–1^, 1540 cm^–1^, 1650 cm^–1^, and 3300 cm^–1^. Carbonate ion substitutes within the crystalline structure were also detected, as indicated by the small single peak at 870 cm^–1^ and the double bands around 1430 cm^–1^ and 1450 cm^–1^, which indicate a type-B carbonated substitution in the PO_4_^3–^ position (Landi et al., 2008; Kourkoumelis and Tzaphlidou, 2010). XRD analysis showed both scaffolds shared a similar profile to that of native bone tissue reported in the literature, specifically large peak at 32 degrees and a smaller peak at 26 degrees (Figure 2C). The Mg/Ca substitution ratio was evaluated to be approximately 4.8 and 4.2% for MgHA and MgHA/pSi scaffolds, respectively (Figure 2D).

### Cell Number and Infiltration in Static and Perfused Conditions

**FIGURE 3:**
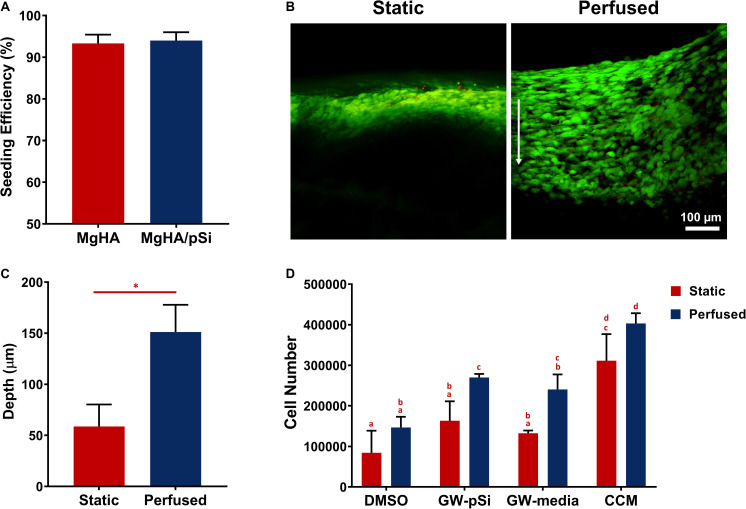
**(A)** hMSCs seeding efficiency on scaffolds (*n* = 3). **(B)** Representative cross-sectional images of live-dead staining of hMSCs after 8 days of culture in MgHA scaffolds in static and perfused conditions, with direction of flow indicated by the white arrow. **(C)** Average depth at which stacked live–dead images decreased to 50% of maximum fluorescence intensity (Student’s *t*-test, *p* < 0.05, *n* = 3, error bars depict standard deviation). **(D)** hMSCs population, as quantified by GAPDH expression via qRT-PCR, shows significant increase in cell number in response to perfusion in various scaffold and culture media combinations. Statistical testing was performed by two-way ANOVA, followed by a Tukey’s multiple comparison between all scaffold/media combinations. Columns with matching letters indicate the comparison was not significant (Student’s *t*-test, ^∗^*p* < 0.05, *n* = 3, error bars depict standard deviation).

A seeding efficiency above 93% was observed for each scaffold composition (Figure 3A). After 8 days of static culture in MgHA scaffold with CCM, live-dead staining revealed closely packed cells near the surface of the scaffolds, with a majority of cells staying within 100 μm from the surface of the scaffolds. In contrast, hMSCs perfused for 8 days invaded more deeply into the scaffold (Figure 3B). Semi-quantitative analysis of confocal images showed a rapid drop in fluorescence in the static samples, with a 50% reduction approximately 60 μm into the scaffold, as compared to 150 μm for perfused scaffolds (Figure 3C).

Next, cell number was quantified by GAPDH expression for the eight culture conditions (Figure 3D). Two-way ANOVA indicated that perfusion increased the average cell number in all scaffolds and a lack of interaction between perfusion and the scaffold/culture media formulation (Supplementary Table 3). Multi-comparison testing indicated that, in perfused cultures, GW9662 administered locally, increased cell number significantly relative to the DMSO control (Supplementary Table 4).

### Microsphere Characterization and *in vitro* Release of GW9662

**FIGURE 4:**
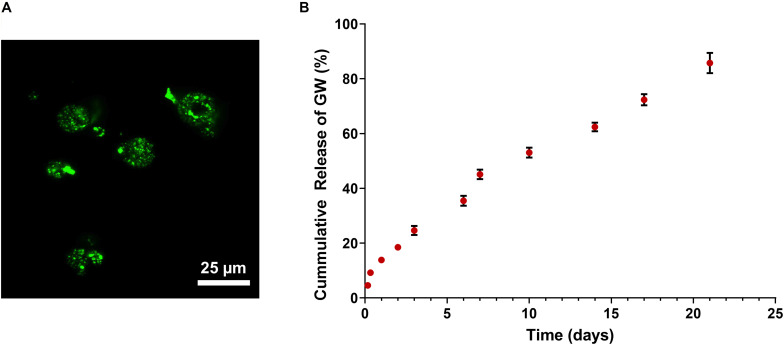
**(A)** Confocal imaging of FITC-labeled PLGA/pSi show pSi are uniformly distributed within PLGA spheres. **(B)** Cumulative release of GW9662 from PLGA/pSi microspheres show controlled release of GW9662 up to 21 days (*n* = 3, error bars depict standard deviation).

Fluorescently labeled pSi were used to characterize their distribution within PLGA microspheres (Figure 4A). Images indicated that the pSi were distributed uniformly within individual microspheres and that pSi content appeared uniform when comparing different PLGA microspheres.

The rate of release of GW9662 from the composite pSi/PLGA microspheres was quantified over a 21-day period since osteogenic differentiation of hMSCs using GW9662 involves continuous application of GW9662 for this duration (Krause et al., 2010). A 14% burst release occurred in the first 24 h, followed by a constant rate of release over the remainder of the experiment, with 82% of the drug released after 21 days (Figure 4B).

### Deposition of Anabolic Bone ECM

It has been previously shown that hMCSs treated with GW9662 secrete an anabolic bone ECM rich in Coll VI and XII, which not only improves retention of hMSCs at an injury site, but also upregulates various osteogenic and angiogenic factors (Clough et al., 2015). To validate the efficacy of the controlled release of GW9662 from PLGA/pSi microspheres and to explore the effects of the fluid shear stress on the expression and deposition of this anabolic bone matrix, qRT-PCR analysis and immunostaining for Coll VI and Coll XII was performed after 8 days of static and perfused culture. The length of culture time was chosen to coincide with the formation of fibrocartilaginous callus seen during fracture repair (Wang and Yeung, 2017).

**FIGURE 5:**
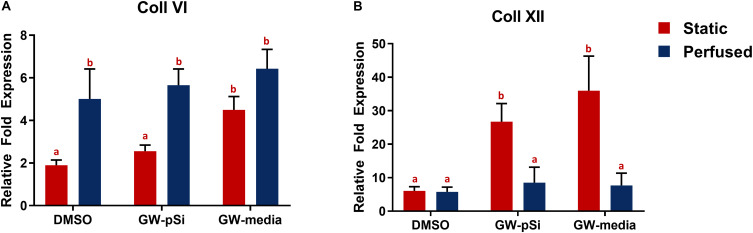
qRT-PCR analysis for the expression of anabolic bone ECM components. **(A)** Coll VI and **(B)** Coll XII relative fold expression in hMSCs after 8 days of culture in various scaffold/media combinations. Columns with matching letters indicate all possible comparisons were not significant (*n* = 3, error bars depict standard deviation, *p* < 0.05, two-way ANOVA, Tukey’s multiple comparison test).

**FIGURE 6:**
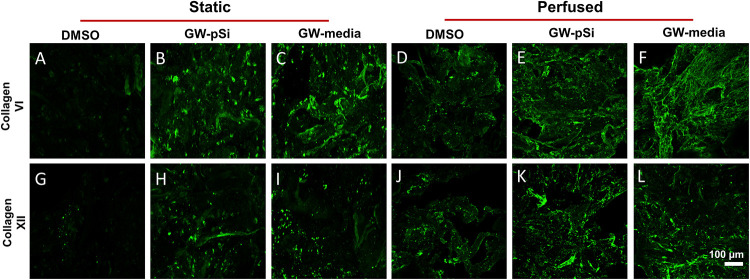
Immunostaining of Coll VI **(A–F)** and XII **(G–L)** after 8 days under static and perfused conditions for various scaffold/culture media combinations reveals similar levels of matrix deposition between GW-pSi and GW-media. Perfusion increased matrix deposition in all scaffold and culture media combinations relative to their static counterparts.

Consistent with previous findings (Clough et al., 2015), hMSCs cultured under static conditions in media supplemented with GW9662 expressed Coll VI in significantly higher amounts than in the absence of GW9662 and cultures in which GW9662 was provided using microspheres (Figure 5A). Interestingly, the expression for Coll VI was increased by perfusion culture for each condition, though this difference was not significant in the cultures in which GW9662 was added directly to the media. Furthermore, perfusion resulted in comparable levels of Coll VI expression between DMOS, GW-pSi, and GW-media scaffolds. The simple interaction—diminishing returns of GW9662-treatment on Coll VI gene expression by perfusion—was confirmed to be significant by two-way ANOVA (Supplementary Table 6). Immunostained samples indicated comparable amounts of Coll VI in the cultures in which GW9662 was administered via controlled release (Figure 6B) or direct addition to the media (Figure 6C), while the Coll VI was barely detectable in the cultures lacking GW9662 (Figure 6A). Perfusion increased amount of Coll VI detected by immunostaining in each condition, with the levels noticeably higher for samples containing GW9662 (Figures 6E,F) than samples lacking GW9662 (Figure 6D).

Under static conditions, Coll XII gene expression was significantly higher in hMSCs cultured in media supplemented with GW9662 than in cultures lacking GW9662 (Figure 5B). In addition, controlled release of GW9662 from microspheres resulted in comparable expression levels to exogeneous GW9662 administration, highlighting the utility of the composite microspheres as an *in situ* drug release platform capable of replacing traditional media treatments. It was also noted that despite the lack of GW9662, Coll XII gene expression was positively expressed in DMSO scaffolds (Figure 5B). Furthermore, in contrast to the trend observed for Coll VI gene expression, Coll XII gene expression either stayed consistent or decreased in response to perfusion, suggesting at a suppressive interaction between perfusion and GW9662 treatment on Coll XII gene expression (Supplementary Table 8). Immunostaining of samples cultured under static conditions confirmed comparable deposition of Coll XII between cultures in which GW9662 was administered directly to the media (Figure 6I) and controlled release (Figure 6H). In contrast, Coll XII deposition was scarce in samples lacking GW9662 (Figure 6G). Perfusion increased the amount of Coll XII detected via immunostaining in all conditions, with noticeably higher depositions in samples treated with GW9662 (Figures 6K,L) than in samples lacking GW9662 (Figure 6J).

### *In vitro* Osteogenic Differentiation in Osteoinductive Scaffolds

**FIGURE 7:**
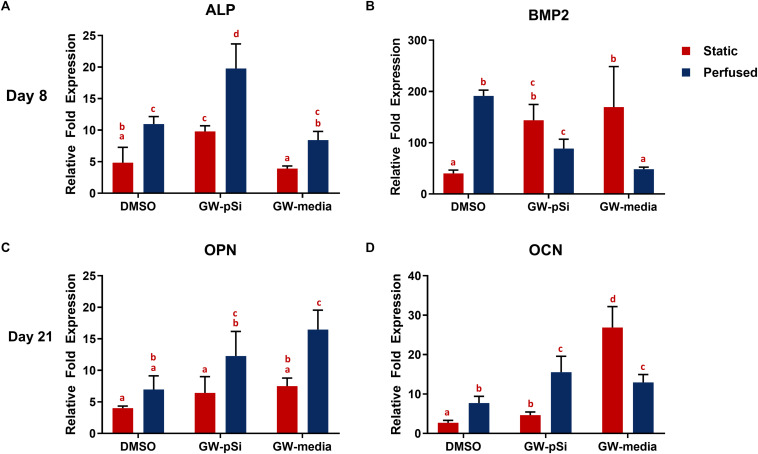
qRT-PCR analysis for the expression of osteogenic markers in hMSCs after **(A,B)** 8 days or **(C,D)** 21 days of culture in various scaffold/media combinations. Fold changes were normalized to the expression levels of uninduced hMSCs after 8 or 21 days of 2D culture. Columns with matching letters indicate all possible comparisons were not significant (*n* = 3, error bars depict standard deviation, *p* < 0.05, two-way ANOVA, Tukey’s multiple comparison test).

To assess the osteoinductive potency of the scaffolds, the relative expression of the early osteogenic markers ALP and BMP2 were examined after 8 days of culture, while the late markers osteocalcin (OCN) and osteopontin (OPN) were investigated after 21 days of culture by qRT-PCR analysis. Briefly, no significant difference was observed in ALP expression between GW-media and DMSO scaffolds under static conditions. In contrast, ALP expression increased significantly in GW-pSi scaffolds in comparison to both the DMSO and GW-media scaffolds (Figure 7A). Perfusion further upregulated ALP expression in all scaffold compositions (Figure 7A). No significant interaction between the effects of perfusion and media/scaffold formulation on ALP expression was observed (Supplementary Table 10). BMP2 gene expression in the absence of perfusion increased in response to GW9662 treatment, with both GW-pSi and GW-media scaffolds having significantly higher expression than the DMSO scaffolds (Figure 7B). A significant increase in BMP2 gene expression was observed in the vehicle controls in response to perfusion compared to its static counterpart. In contrast, BMP2 gene expression was negatively impacted in both exogenous and drug-eluting GW9662 samples under perfusion (Figure 7B). The interaction between perfusion and media/scaffold formulation was found to be significant (Supplementary Table 12). To further investigate these trends at the protein level, immunoblotting against BMP2 was performed. Immunoblotting revealed GW9662 treatment and perfusion had a synergistic effect on BMP2 expression at the translational level (Supplementary Figure 2).

A trademark of terminally differentiated hMSCs is the expression of late osteogenic markers. Thus, expression of osteopontin (OPN) and osteocalcin (OCN) at the transcription level was investigated after 21 days of culture. OPN was positively expressed in all scaffolds and media conditions, with the lowest level expressed in the DMSO scaffolds (Figure 7C). Although OPN expression increased in both the exogeneous and GW9662-eluting microspheres scaffolds relative to the DMSO control under static conditions, the trend was not significant. Perfusion significantly upregulated OPN expression in both GW-pSi and GW-media scaffolds relative to the static DMSO scaffolds. The interaction between perfusion and scaffold/media formulation and their effects on OPN expression was not significant (Supplementary Table 14). OCN expression was positively expressed in DMSO scaffolds and responded strongly in a dose-depended manner to GW9662 treatment. Briefly, GW-media and GW-pSi had significantly higher levels of expression relative to the DMSO control (Figure 7D). The application of perfusion stimulated higher levels of OCN expression in GW-pSi and DMSO scaffolds while decreasing OCN expression in GW-media scaffolds. However, fold expression in perfused GW-media scaffolds were still significantly higher than the static vehicle control. The interaction between perfusion and scaffold/media formulation on OCN was found to be significant (Supplementary Table 16).

### *In vitro* Mineralization and Scaffold Compressive Strength

**FIGURE 8:**
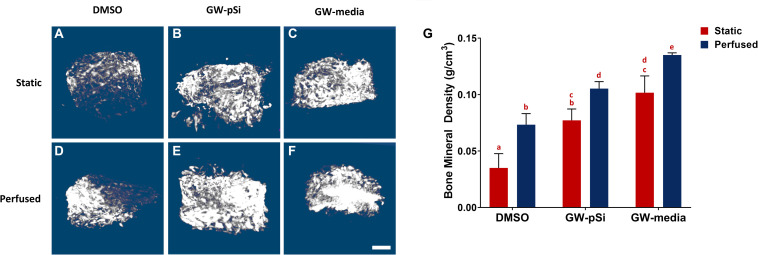
Representative μCT reconstructions of scaffolds after 21 days of culture **(A–F)** (scale bar 500 μm). **(G)** Bone mineral density increases in response to both perfusion and GW9662. Columns with matching letters indicate all possible comparisons were not significant (*n* = 3, error bars depict standard deviation, *p* < 0.05, two-way ANOVA, Tukey’s multiple comparison test).

Mineralization within the scaffolds was detected with μCT. The degree of radio-opacity of reconstructed μCT images of the scaffolds harvested after 21 days of culture (Figures 8A–F) is summarized in Figure 8G. DMSO (Figure 8A) scaffolds cultured under static conditions were largely radiolucent, while larger volume of GW-pSi (Figure 8B) and GW-media (Figure 8C) scaffolds were detected. Furthermore, denser regions, which correlate to degree of mineralization, were detected in all perfused samples, with larger regions detected in the GW-pSi (Figure 8E) and GW-media (Figure 8F) scaffolds. When quantified using the attenuation coefficient method, significantly higher levels in bone mineral density were detected in scaffolds cultured under perfusion relative to their static counterparts in all three scaffolds/media conditions (Figure 8G). The interaction between perfusion and scaffold/media formulation and their effects on BMD was not significant (Supplementary Table 19). Under static conditions, GW9662 treatment resulted in significantly higher BMD levels compared to the DMSO scaffolds, regardless of GW-source. A similar trend was observed under perfusion (Figure 8G).

**FIGURE 9:**
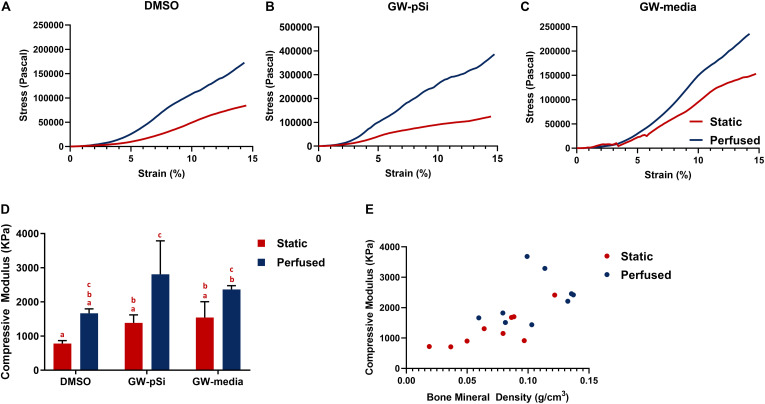
**(A–C)** Representative compressive tests of scaffolds after 21 days of culture. **(D)** Average compressive modulus of scaffolds after 21 days of culture. Columns with matching letters indicate all possible comparisons were not significant (*n* = 3, error bars depict standard deviation, *p* < 0.05, two-way ANOVA, Tukey’s multiple comparison test). **(E)** High degree of correlation between observed bone mineral density and compressive modulus for the compiled 18 samples (Pearson’s correlation *r* value of 0.695, *p* < 0.05).

We then evaluated whether the augmented bone mineral density translated to improved mechanical properties. Although the variances in scaffold compressive moduli were not homogenous due in part to an outlier in GW-pSi perfused samples, a two-way ANOVA and Tukey’s multiple comparison test was still performed to ensure similar analysis could be performed between assays (Supplementary Table 21). Mechanical testing revealed a general trend in improved compressive modulus in response to perfusion (Figures 9A–C). Although the compressive modulus increased in response to perfusion in DMSO and GW-media scaffolds in comparison to their static counterparts, the trend was not significant (Figure 9D). In contrast, a significant difference was observed between perfused and static GW-pSi scaffolds (Figure 9D). Furthermore, the combined treatment of GW9662, through either exogenous means or microspheres, and perfusion resulted in compressively stiffer scaffolds compared to the static MgHA/DMSO scaffold (Figure 9D). Lastly, a strong correlation between bone mineral density and compressive modulus was observed for the 18 pooled samples, with a Person’s correlation *r* value of 0.696 and a two-tailed *p*-value of 0.014 (Figure 9E).

## Discussion

Several key morphogenetic pathways, e.g., Wnts, BMPs, active during embryonic skeletal development are observed during bone repair, allowing bone tissue to be restored to its uninjured state (Bais et al., 2009; Einhorn and Gerstenfeld, 2015). During the anabolic phase of bone repair, a callus tissue is deposited by recruited progenitor cells and is partially mineralized (Little et al., 2007; Einhorn and Gerstenfeld, 2015). This anabolic bone tissue is characterized by high levels of unique types of collagens, such as Coll VI and XII, that play a major regulatory role (Izu et al., 2011; Kohara et al., 2016). Furthermore, nascent ossified bone tissue in both fracture calluses and in developing bone contain ionic substitutions (Quint et al., 1980; Kakei et al., 1997), specifically Mg^2+^, in the hydroxyapatite crystalline structure that allows for rapid ion exchange. Thus, the key to the development of new scaffolds for bone repair lies in recapitulating both the inorganic and organic phases of the anabolic bone niche. The response of hMSCs cultured in a macroporous Coll/MgHA scaffold with GW9662-eluting microspheres for the purpose of depositing an ECM rich in Coll VI and XII demonstrates the feasibility of producing a scaffold that resembles both mineral and collagen content of regenerating bone.

The fabrication of the scaffolds was inspired by the biomineralization process of trabecular bone, which can be described as a poorly crystalline biological apatite with ionic substitutions coating an underlying macroporous protein structure (Landi et al., 2008; Figueiredo et al., 2012). Through controlled freezing and lyophilization, it was possible to fabricate a highly porous composite scaffold, which lends itself to high cell infiltration when coupled with a perfusion bioreactor. Upon evaluation, the scaffolds showed a close similarity in their morphology and mineral composition to that of trabecular bone tissue. Briefly, the specific bone surface ratio (BS/BV) of our scaffolds (*cf*. Figure 1D) was similar to values (17.03 ± 4.06 mm^–1^) reported for trabecular bone in the human femur (Teo et al., 2006). Analysis of the scaffolds’ composition revealed additional substitutions in the apatite structure other than Mg^2+^ substituting for Ca^2+^. Carbonate ion substitutes, specifically type-B (870 cm^–1^), were also detected within the lattice structure. The presence of Mg^2+^ and carbonate ions are particular noteworthy as they are typically found in younger, more reactive bone, as they allow for reversible exchange of ions (Landi et al., 2008; Rey et al., 2009; Figueiredo et al., 2012), which is lost as bone matures. Thus, the presence of Mg^2+^ and type-B carbonate ions reaffirms that the scaffolds were effectively recapitulating the mineral features of immature bone, a highly osteogenic environment.

Although the fabricated scaffolds mimicked key mineral features of newly formed bone, its sole use of Coll I in the underlying protein structure fails to mimic the cocktail of rare collagens that are upregulated in developing and regenerating bone. Specifically, Coll XII is abundantly present in the outer and inner layers of the periosteum of developing long bones (Wälchli et al., 1994), and has been shown to play an important regulatory role in osteoblast differentiation and bone matrix formation (Izu et al., 2011). Coll VI is highly expressed in the primary osteons of the femoral diaphysis, where it supports the proliferation and maturation of pre-osteoblasts (Kohara et al., 2015). Coll VI is also highly expressed in the middle layer of the Groove of Ranvier, and important ossifying region in long bone development, and helps regulate the differentiation of pre-osteoblasts prior to maturation (Kohara et al., 2016). We have previously shown that hMSCs treated with GW9662 secrete a complex cocktail of ECM that contains high levels of Coll VI and Coll XII, which can be used to extend the retention of hMSCs at a lesion site, resulting in superior bone healing (Zeitouni et al., 2012). Although the ECM generated from GW9662-treated hMSCs have shown a remarkable capacity to regenerate bone even when administered to lesion sites without hMSCs, its purification and fabrication into a 3D scaffold is an expensive, time-consuming process. Stimulating hMSCs to deposit the anabolic bone ECM on established scaffolds after implantation would circumvent some of these challenges and facilitate translation to the clinic. However, GW9662 necessitates constant replenishing to achieve its desired effects due to a short half-life (Li et al., 2011). We hypothesized that incorporating a drug delivery system capable of controlled release of GW9662 into porous mineralized scaffold would yield comparable deposition of ECM compared to traditional fabrication methods (10 μM every 2–3 days). Although direct implantation of hMSCs cultured on scaffolds embedded with GW9662-eluting microspheres is a possible clinical application route, we also explored the use of a perfusion bioreactor to improve deposition of the anabolic bone ECM throughout the scaffold. Together, the drug eluting microspheres and the perfusion bioreactor could be utilized to fabricate clinically relevant scaffolds for bone repair.

In order to fully understand Coll VI and Coll XII deposition trends, gene expression was correlated with immunostaining. While mRNA expression of Coll VI and Coll XII increased to varying degrees due to GW9662 and the drug eluting microspheres, only Coll VI expression increased in response to perfusion. In fact, a decrease in Coll XII expression was observed after 8 days of culture in the bioreactor. Immunostaining, however, showed a striking difference between static and perfused samples, revealing a remarkable increase in deposition of both Coll VI and Coll XII in terms of uniformity and density throughout GW-pSi and GW-media scaffolds. This result is at least partially attributable to the relatively high amount of hMSCs and their distribution deeper into the scaffold. Increased ECM deposition by hMSCs due to fluid shear stress has been previously reported (Grayson et al., 2011), suggesting another mechanism by which perfusion promotes ECM deposition in the scaffolds. To our knowledge, this is the first time that perfusion has been used to in conjunction with a drug eluting system to fabricate a unique blend of matrix proteins for the purpose of mimicking the complex microenvironment of anabolic bone. Furthermore, the GW9662 eluting microspheres were capable of stimulating the deposition of the anabolic bone ECM using significantly less GW9662 compared to exogenously supplemented culture media; 0.52 μg of GW9662 in GW-pSi for 8 days of culture vs. 5.5 μg GW9662 added every 2 days in GW-media scaffolds.

As trabecular bone is major reservoir of progenitor cells (Sakaguchi et al., 2004), we initially quantified hMSC infiltration into the scaffolds under both static and perfused conditions without the influence of GW9662. In agreement with previous studies, cells cultured under static conditions form a dense layer constrained to the scaffold surface (Grayson et al., 2008; Correia et al., 2013). Perfusion of cells cultured in CCM drove the cells to distribute more uniformly into the scaffold, but did not significantly increase total cell number, suggesting that the enhanced distribution of the cells was primarily due to enhanced cell migration into the scaffold. With the exception of scaffolds cultured in CCM, the calculated cell population in scaffolds cultured under static conditions were lower than the initial seeding number. However, cultivation in the perfusion bioreactors was able to rescue cell number in DMSO, GW-pSi, GW-media scaffolds. A greater commitment toward the osteogenic lineage has been associated with a reduction in hMSCs proliferation rates, which could explain the reason DMSO, GW-pSi, and GW-media scaffolds had lower cell populations than the scaffolds cultured in CCM (Banfi et al., 2002; Wagner et al., 2008; Bara et al., 2014). Furthermore, insufficient gas and nutrient transport during the initial overnight static culture of the perfused samples is likely responsible for cell number being lower than the initial seeding number in DMSO, GW-pSi, and GW-media scaffolds.

The anabolic bone ECM derived from GW9662 treated hMSCs has been shown to upregulate the expression of osteogenic markers and improve bone repair in murine critical size-defect models (Clough et al., 2015, 2017). The use of GW9662-eluting microspheres and perfusion bioreactors to prepare scaffolds with the anabolic bone ECM provides important information to inform translation of this technology to the clinic. We hypothesized that the controlled release of GW9662 and the subsequent deposition of ECM would result in improved osteogenic differentiation of seeded hMSCs. Consistent with previous *in vivo* work (Clough et al., 2015), we observed BMP2 gene expression increase in response to GW9662 (exogenous and from microspheres) in static conditions. Although BMP2 gene expression was lower in GW9662-treated samples under perfused conditions, immunoblotting revealed a synergistic relationship between perfusion and GW9662 treatment. ALP expression in response to GW9662 treatment has been previously shown to be biphasic, becoming inhibitory at higher GW9662 concentrations (Krause et al., 2010). Thus, it was unsurprising to see GW-pSi samples having a higher ALP expression than both GW-media and DMSO scaffolds under static conditions. Although the static DMSO and GW-media scaffolds had comparable levels of ALP expression, both were still positively expressed. Furthermore, application of fluid shear stress significantly increased ALP expression in all three scaffolds. Similarly, gene expression levels for OPN and OCN increased in response to perfused culture in GW-pSi and DMSO scaffolds. Expression of these two late osteogenic markers in perfused GW-media scaffolds was more complex. While OPN expression benefited from perfusion, OCN expression decreased. However, it should be noted that OCN expression in perfused GW-media scaffolds were still highly expressed. For Coll VI, Coll XII, BMP2, and OCN, the interaction was found to be significant, thus the effects of either variable depended on the other. Specifically, the upregulation of Coll VI, Coll XII, and BMP2 expression by GW9662 under static conditions were diminished under perfusion (*cf* Figures 5, 6A,B). When investigated further at the protein level, however, Coll VI, Coll XII, and BMP2 expression was augmented by the dual treatment of GW9662 and perfusion. The mismatch in expression between the transcription and protein level highlights the limitations of utilizing gene expression data in understanding the interaction between perfusion and scaffold/culture media formulations.

The mechanism by which the GW9662-induced anabolic bone ECM mediates the upregulation of BMP2 and other osteogenic markers is not entirely understood, nor is the influence of perfusion. Previous work, however, has shown that signal transduction in hMSCs treated with GW9662 is dominated by integrins and, presumably, their interaction with the newly deposited ECM (Clough et al., 2015). In rodents, Coll VI has been shown to mediate osteoblast differentiation via the cell-surface receptor neural/glial antigen 2 (NG2) and several types of integrins (Kohara et al., 2015, 2016). Coll XII is crucial in regulating osteoblast polarization and cell–cell interaction (Izu et al., 2011). As others have shown, integrin activated MAPK/ERK plays a crucial role in BMP2 induction (Lu and Zreiqat, 2010), and it is feasible to assume a similar mechanism is responsible for the osteoinductive properties of the anabolic bone ECM. Integrins (β1) and other adaptor proteins have also been implicated in mediating hMSCs response to mechanical stimulation (Chen and Jacobs, 2013). For example, application of fluid shear stress has been shown to increase ALP activity and expression of osteogenic markers, along with activation of Focal Adhesion Kinase (FAK) (Chen and Jacobs, 2013). In addition, Coll XII gene expression can be directly stimulated by mechanical strain (Arai et al., 2008). As a PPARγ inhibitor, GW9662 has been shown to upregulate various early stage markers of osteogenesis by attenuating the negative crosstalk on cWnt signaling, allowing for the translocation of β-catenin to the nucleus (Chen and Jacobs, 2013). Although there are multiple mechanotransduction pathways that mediate osteogenic differentiation, exposure to fluid shear stress can also result in the translocation of β-catenin (Chen et al., 2019) to the nucleus in hMSCs mediated by *N*-cadherin signaling rather than cWnt (Arnsdorf et al., 2009). However, these studies are typically performed in a 2D system, highlighting the need to investigate and decouple stimuli stemming from osteogenic niche environments such as from the anabolic bone ECM presented here from mechanical stimuli. At this current time, it is unknown whether cell-to-cell (*N*-cadherin) or cell-to-ECM (integrin) signaling pathways dominate in our system, which combines fluid shear stress from the perfusion bioreactor through a 3D environment mimicking the ECM of anabolic bone, or how the indirect upregulation of cWnt via PPARγ inhibition fits into the complex adhesive signaling networks.

Given the critical function of ALP and OPN in mineralization and the potent ability of BMP2 to induce ectopic bone formation, we evaluated whether their favorable response to GW9662-treatment and perfusion were associated with a similar increase in mineralization. While the microstructure of as-prepared scaffolds could be discerned, their bone mineral density could not be evaluated as they were below the contrast limits of the calibration phantoms. After 3 weeks of culture, however, the bone mineral densities of all scaffolds were within the detection limit. In agreement with the gene expression results, we found significantly elevated BMD levels in the GW-pSi and GW-media scaffolds relative to the DMSO scaffolds. Perfusion augmented BMD levels in DMSO, GW-pSi, and GW-media scaffolds. In addition, the exogenous GW9662 treated scaffolds were mineralized to a significantly higher degree than even the GW-pSi scaffolds under perfusion, further corroborating the existence of synergistic relationship between GW9662-treatment and perfusion. Exemplifying a structure-function relationship, the compressive mechanical properties of bone are highly correlated with mineral density (Follet et al., 2004; Teo et al., 2006). Given that restoring weight-bearing functionality to damaged or diseased bone is a goal of bone tissue engineering, it was crucial to evaluate whether the augmented bone mineral density observed in the cultured scaffolds translated to improved mechanical properties. While perfusion and GW9662 treatment individually improved the compressive properties of the scaffolds, together they resulted in a significantly stiffer scaffold. The compressive moduli were still substantial lower than those reported for native bone, but that is to be expected for experiments representing early mineralization of the scaffolds.

## Conclusion

Despite their limited availability and association with donor site morbidity, autologous bone grafts remain the gold standard treatment for bone repair. Engineering bone constructs large enough for clinical utility is a challenging goal, due in part to our incomplete understanding of the combined effects of soluble factors and fluid shear stress on bone development. In this study, we examined the influence of controlled release of GW9662, a PPARγ inhibitor, and perfusion on the deposition of an ECM rich in collagens characteristic of newly formed bone by human mesenchymal stem cells cultured on osteoinductive scaffold. A striking improvement in deposition of the anabolic bone ECM relative static and vehicle controls showed a synergistic relationship between perfusion and GW9662 treatment, from either the drug-eluting microspheres or media supplementation, resulting in a scaffold that recapitulated both the inorganic and organic fraction of nascent bone. Future *in vivo* work is needed to determine whether Coll/MgHA scaffolds embedded with GW9662-eluting microspheres can incite native osteoprogenitors cells to deposit the anabolic bone ECM at levels that result in improved bone healing. In addition, the data presented here suggests that GW9662 treatment and a perfusion culture system can be utilized to deposit the anabolic bone ECM to augment the osteoregenerative properties of existing scaffolds.

## Data Availability Statement

All datasets generated for this study are included in the article/Supplementary Material.

## Author Contributions

RK, CG, and ET were involved in the conception of the work. FT and SM fabricated the scaffolds for this work. EM was involved in the experimental design, data collection and analysis, with assistance from MC. CG and RK contributed to the data interpretation. EM and RK were involved in drafting the manuscript. All authors contributed to the article and approved the submitted version.

## Conflict of Interest

The authors declare that the research was conducted in the absence of any commercial or financial relationships that could be construed as a potential conflict of interest.
