# Supplementary material for: Mimicking the Organic and Inorganic Composition of Anabolic Bone Enhances Human Mesenchymal Stem Cell Osteoinduction and Scaffold Mechanical Properties
Source: Front Bioeng Biotechnol. 2020 Jul 3;8:753. doi: 10.3389/fbioe.2020.00753 (PMC7347795; doi:10.3389/fbioe.2020.00753)
Supplement: Supplementary file 1 [file Presentation_1.pdf]

Figure Supplementary 1: (A) Schematic illustrating the fabrication of PLGA/pSi microspheres in Coll/MgHA scaffolds. (B) Cross sectional diagram indicates culture media flows from bottom to top through a porous scaffold, as indicated by the arrows. (C) Top view of the perfusion bioreactor, which is capable of culturing six constructs simultaneously.

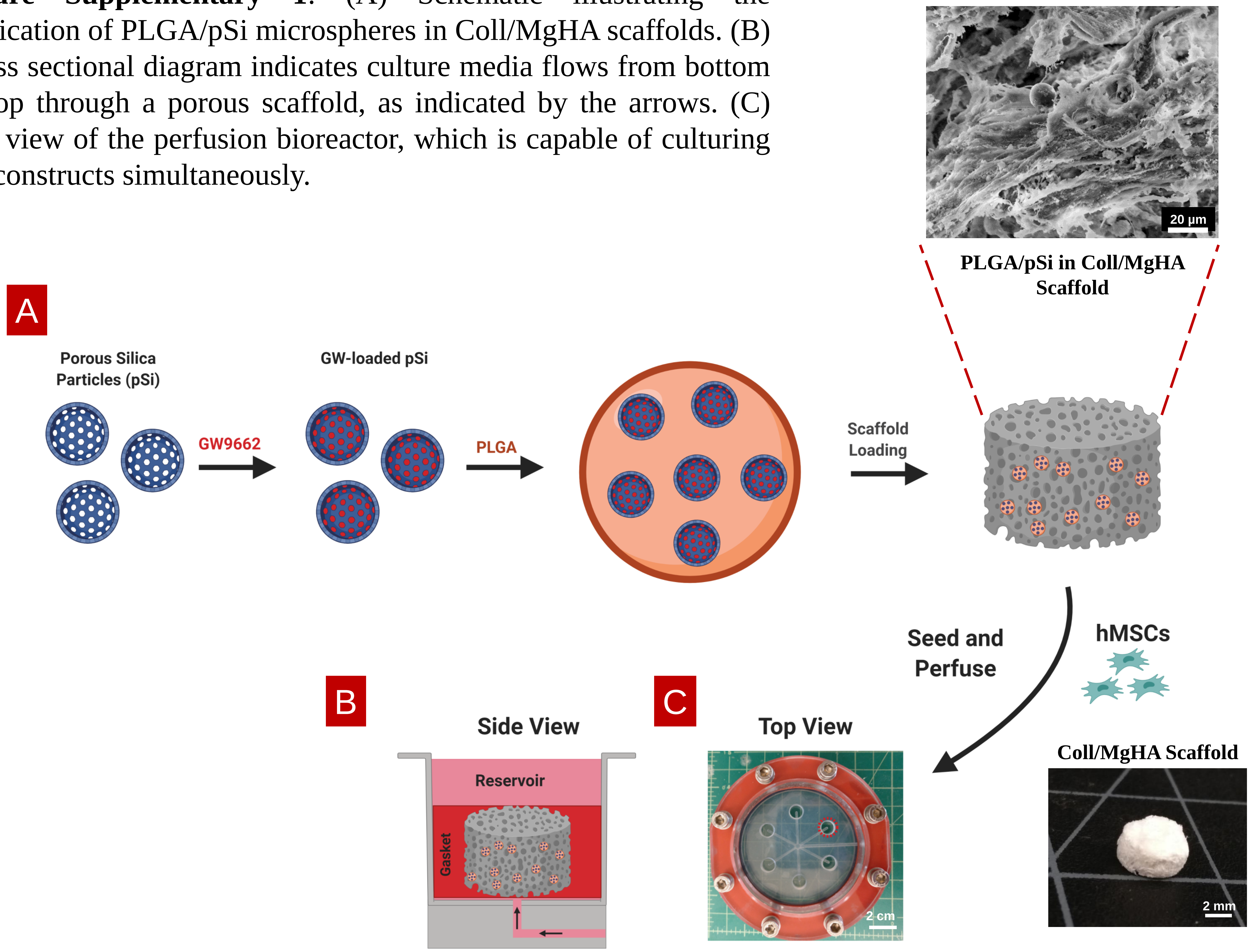

Target	Sequence
Human GAPDH	Forward: ctctctgctcctcctgttcgac<br>Reverse: tgagcgatgtggctcggct
Human Collagen VI	Forward: ccatcgtgcgcagcc<br>Reverse: tgcgccgactcgtgc
Human Collagen XII	Forward: cttccattgaggcagaagtt<br>Reverse: agacacaagagcagcaatga
Human BMP2	Forward: cccagcgtgaaaagagagac<br>Reverse: gagaccgcagtccgtctaag
Human ALP	Forward: gacccttgacccccacaat<br>Reverse: gctcgtactgcatgtcccct
Human OCN	Forward: tcacactcctcgccctattg<br>Reverse: ctcttcactacctcgctgcc
Human OPN	Forward: catcacctgtgccataccagtt<br>Reverse: ttggaagggtctgtggggcta

Table Supplementary 1: Primer sequences utilized for qRT-PCR analysis.

Scaffold/Culture Condition	DF	Variance	χ^2	P Value
CCM/Static	7	6.44E+09	10.7985	0.14765
DMSO/Static		4.45E+09		
pSi/Static		3.45E+09		
GW/Static		6.57E+07		
CCM/Perfused		9.40E+08		
DMSO/Perfused		1.03E+09		
pSi/Perfused		1.19E+08		
GW/Perfused		2.05E+09		

Table Supplementary 2: Bartlett's test for homogeneity of variances on calculated cell number for all scaffold and media formulations.

ANOVA table	SS	DF	MS	F (DFn, DFd)	P value
Interaction	2015562891	3	671854297	F (3, 16) = 0.4348	P=0.7311
Scaffold/Culture Media	185357046568	3	61785682189	F (3, 16) = 39.99	P<0.0001
Perfusion	51029447337	1	51029447337	F (1, 16) = 33.03	P<0.0001
Residual	24722643661	16	1545165229		

Table Supplementary 3: Two-way ANOVA table for the calculated cell number for all scaffold and media formulations.

Tukey's multiple comparisons test	Mean 1	Mean 2	Mean Diff.	95.00% CI of diff.	Adjusted P Value
DMSO:Static vs. DMSO:Perfused	84268	146683	-62415	-173534 to 48704	0.5430
DMSO:Static vs. pSi:Static	84268	163278	-79010	-190129 to 32109	0.2778
DMSO:Static vs. pSi:Perfused	84268	269831	-185563	-296682 to -74444	0.0006
DMSO:Static vs. GW:Static	84268	132434	-48166	-159285 to 62953	0.7966
DMSO:Static vs. GW:Perfused	84268	240461	-156193	-267312 to -45074	0.0033
DMSO:Static vs. CCM:Static	84268	311292	-227024	-338143 to -115905	<0.0001
DMSO:Static vs. CCM:Perfused	84268	403185	-318917	-430036 to -207799	<0.0001
DMSO:Perfused vs. pSi:Static	146683	163278	-16595	-127714 to 94524	0.9994
DMSO:Perfused vs. pSi:Perfused	146683	269831	-123148	-234266 to -12029	0.0245
DMSO:Perfused vs. GW:Static	146683	132434	14249	-96870 to 125368	0.9998
DMSO:Perfused vs. GW:Perfused	146683	240461	-93778	-204896 to 17341	0.1325
DMSO:Perfused vs. CCM:Static	146683	311292	-164609	-275727 to -53490	0.0020
DMSO:Perfused vs. CCM:Perfused	146683	403185	-256502	-367621 to -145383	<0.0001
pSi:Static vs. pSi:Perfused	163278	269831	-106553	-217672 to 4566	0.0651
pSi:Static vs. GW:Static	163278	132434	30844	-80275 to 141963	0.9739
pSi:Static vs. GW:Perfused	163278	240461	-77183	-188302 to 33936	0.3020
pSi:Static vs. CCM:Static	163278	311292	-148014	-259133 to -36895	0.0054
pSi:Static vs. CCM:Perfused	163278	403185	-239907	-351026 to -128789	<0.0001
pSi:Perfused vs. GW:Static	269831	132434	137397	26278 to 248515	0.0104
pSi:Perfused vs. GW:Perfused	269831	240461	29370	-81749 to 140489	0.9800
pSi:Perfused vs. CCM:Static	269831	311292	-41461	-152580 to 69658	0.8892
pSi:Perfused vs. CCM:Perfused	269831	403185	-133355	-244473 to -22236	0.0132
GW:Static vs. GW:Perfused	132434	240461	-108027	-219145 to 3092	0.0598
GW:Static vs. CCM:Static	132434	311292	-178858	-289976 to -67739	0.0009
GW:Static vs. CCM:Perfused	132434	403185	-270751	-381870 to -159632	<0.0001
GW:Perfused vs. CCM:Static	240461	311292	-70831	-181950 to 40288	0.3967
GW:Perfused vs. CCM:Perfused	240461	403185	-162725	-273843 to -51606	0.0022
CCM:Static vs. CCM:Perfused	311292	403185	-91894	-203012 to 19225	0.1464

Table Supplementary 4: Tukey-multiple comparison results between calculated cell number for all scaffold and media formulations.

Gene	DF	Variances of Ct							χ^2	P Value
		CCM Monolayer	DMSO Static	pSi Static	GW Static	DMSO Perfused	pSi Perfused	GW Perfused		
GAPDH (Day 8)	6	0.040915	0.043722	0.012726	0.008238	0.027559	0.001815	0.00081	8.4176	0.20905
Coll VI (Day 8)	6	0.091026	0.003855	0.022544	0.04407	0.048581	0.187793	0.04922	5.69369	0.45836
Coll XII (Day 8)	6	0.925951	0.062559	0.094248	0.190015	0.151693	0.580326	0.47209	4.76457	0.57434
ALP (Day 8)	6	0.109881	0.053793	0.010559	0.008893	0.027848	0.030492	0.05182	3.81929	0.70111
BMP2 (Day 8)	6	0.332015	0.026678	0.104059	0.4476	0.004559	0.15927	0.017878	10.3546	0.11049
GAPDH (Day 21)	6	0.058337	0.000144	0.040959	0.041181	0.098233	0.006381	0.026181	10.8626	0.09272
OPN (Day 21)	6	0.006004	0.021264	0.320164	0.035093	0.118517	0.229544	0.071987	7.76493	0.25584
OCN (Day 21)	6	1.578033	0.129744	1.605737	0.060859	0.146293	0.15957	0.037544	11.1802	0.08296

Table Supplementary 5: Bartlett's test for homogeneity of variances for the C_T values for all genes/times points.

ANOVA table	SS	DF	MS	F (DFn, DFd)	P value
Interaction	0.6261	2	0.3131	F (2, 12) = 6.567	P=0.0118
Presence/Source of GW	1.981	2	0.9903	F (2, 12) = 20.77	P=0.0001
Perfusion	4.688	1	4.688	F (1, 12) = 98.34	P<0.0001
Residual	0.5720	12	0.04767		

Table Supplementary 6: Two-way ANOVA table results for the Coll VI $\Delta\Delta C_T$ values.

Tukey's multiple comparisons test	Mean 1	Mean 2	Mean Diff.	95.00% CI of diff.	Adjusted P Value
DMSO:Static vs. DMSO:Perfused	-0.9200	-2.326	1.406	0.8072 to 2.005	<0.0001
DMSO:Static vs. pSi:Static	-0.9200	-1.360	0.4400	-0.1588 to 1.039	0.1951
DMSO:Static vs. pSi:Perfused	-0.9200	-2.500	1.580	0.9812 to 2.179	<0.0001
DMSO:Static vs. GW:Static	-0.9200	-2.170	1.250	0.6512 to 1.849	0.0001
DMSO:Static vs. GW:Perfused	-0.9200	-2.686	1.766	1.167 to 2.365	<0.0001
DMSO:Perfused vs. pSi:Static	-2.326	-1.360	-0.9660	-1.565 to -0.3672	0.0014
DMSO:Perfused vs. pSi:Perfused	-2.326	-2.500	0.1740	-0.4248 to 0.7728	0.9115
DMSO:Perfused vs. GW:Static	-2.326	-2.170	-0.1560	-0.7548 to 0.4428	0.9417
DMSO:Perfused vs. GW:Perfused	-2.326	-2.686	0.3600	-0.2388 to 0.9588	0.3691
pSi:Static vs. pSi:Perfused	-1.360	-2.500	1.140	0.5412 to 1.739	0.0003
pSi:Static vs. GW:Static	-1.360	-2.170	0.8100	0.2112 to 1.409	0.0060
pSi:Static vs. GW:Perfused	-1.360	-2.686	1.326	0.7272 to 1.925	<0.0001
pSi:Perfused vs. GW:Static	-2.500	-2.170	-0.3300	-0.9288 to 0.2688	0.4549
pSi:Perfused vs. GW:Perfused	-2.500	-2.686	0.1860	-0.4128 to 0.7848	0.8873
GW:Static vs. GW:Perfused	-2.170	-2.686	0.5160	-0.08280 to 1.115	0.0990

Table Supplementary 7: Tukey-multiple comparison results between $\Delta\Delta C_T$ for Coll VI.

ANOVA table	SS	DF	MS	F (DFn, DFd)	P value
Interaction	3.739	2	1.869	F (2, 12) = 10.36	P=0.0024
Presence/Source of GW	8.110	2	4.055	F (2, 12) = 22.47	P<0.0001
Perfusion	7.805	1	7.805	F (1, 12) = 43.24	P<0.0001
Residual	2.166	12	0.1805		

Table Supplementary 8: Two-way ANOVA table results for the Coll XII $\Delta\Delta C_T$ values.

Tukey's multiple comparisons test	Mean 1	Mean 2	Mean Diff.	95.00% CI of diff.	Adjusted P Value
DMSO:Static vs. DMSO:Perfused	-2.602	-2.530	-0.07200	-1.237 to 1.093	>0.9999
DMSO:Static vs. pSi:Static	-2.602	-4.740	2.138	0.9729 to 3.303	0.0005
DMSO:Static vs. pSi:Perfused	-2.602	-3.090	0.4880	-0.6771 to 1.653	0.7230
DMSO:Static vs. GW:Static	-2.602	-5.169	2.567	1.402 to 3.732	<0.0001
DMSO:Static vs. GW:Perfused	-2.602	-2.940	0.3380	-0.8271 to 1.503	0.9176
DMSO:Perfused vs. pSi:Static	-2.530	-4.740	2.210	1.045 to 3.375	0.0004
DMSO:Perfused vs. pSi:Perfused	-2.530	-3.090	0.5600	-0.6051 to 1.725	0.6052
DMSO:Perfused vs. GW:Static	-2.530	-5.169	2.639	1.474 to 3.804	<0.0001
DMSO:Perfused vs. GW:Perfused	-2.530	-2.940	0.4100	-0.7551 to 1.575	0.8371
pSi:Static vs. pSi:Perfused	-4.740	-3.090	-1.650	-2.815 to -0.4849	0.0048
pSi:Static vs. GW:Static	-4.740	-5.169	0.4290	-0.7361 to 1.594	0.8114
pSi:Static vs. GW:Perfused	-4.740	-2.940	-1.800	-2.965 to -0.6349	0.0024
pSi:Perfused vs. GW:Static	-3.090	-5.169	2.079	0.9139 to 3.244	0.0007
pSi:Perfused vs. GW:Perfused	-3.090	-2.940	-0.1500	-1.315 to 1.015	0.9976
GW:Static vs. GW:Perfused	-5.169	-2.940	-2.229	-3.394 to -1.064	0.0004

Table Supplementary 9: Tukey-multiple comparison results between $\Delta\Delta C_T$ for Coll XII.

ANOVA table	SS	DF	MS	F (DFn, DFd)	P value
Interaction	0.02014	2	0.01007	F (2, 12) = 0.1161	P=0.8914
Presence/Source of GW	5.275	2	2.638	F (2, 12) = 30.41	P<0.0001
Perfusion	5.438	1	5.438	F (1, 12) = 62.71	P<0.0001
Residual	1.041	12	0.08673		

Table Supplementary 10: Two-way ANOVA table results for the ALP $\Delta\Delta C_T$ values.

Tukey's multiple comparisons test	Mean 1	Mean 2	Mean Diff.	95.00% CI of diff.	Adjusted P Value
DMSO:Static vs. DMSO:Perfused	-2.278	-3.454	1.176	0.3683 to 1.984	0.0039
DMSO:Static vs. pSi:Static	-2.278	-3.294	1.016	0.2083 to 1.824	0.0116
DMSO:Static vs. pSi:Perfused	-2.278	-4.307	2.029	1.221 to 2.837	<0.0001
DMSO:Static vs. GW:Static	-2.278	-1.964	-0.3140	-1.122 to 0.4937	0.7769
DMSO:Static vs. GW:Perfused	-2.278	-3.073	0.7950	-0.01267 to 1.603	0.0546
DMSO:Perfused vs. pSi:Static	-3.454	-3.294	-0.1600	-0.9677 to 0.6477	0.9826
DMSO:Perfused vs. pSi:Perfused	-3.454	-4.307	0.8530	0.04533 to 1.661	0.0364
DMSO:Perfused vs. GW:Static	-3.454	-1.964	-1.490	-2.298 to -0.6823	0.0005
DMSO:Perfused vs. GW:Perfused	-3.454	-3.073	-0.3810	-1.189 to 0.4267	0.6224
pSi:Static vs. pSi:Perfused	-3.294	-4.307	1.013	0.2053 to 1.821	0.0118
pSi:Static vs. GW:Static	-3.294	-1.964	-1.330	-2.138 to -0.5223	0.0014
pSi:Static vs. GW:Perfused	-3.294	-3.073	-0.2210	-1.029 to 0.5867	0.9341
pSi:Perfused vs. GW:Static	-4.307	-1.964	-2.343	-3.151 to -1.535	<0.0001
pSi:Perfused vs. GW:Perfused	-4.307	-3.073	-1.234	-2.042 to -0.4263	0.0026
GW:Static vs. GW:Perfused	-1.964	-3.073	1.109	0.3013 to 1.917	0.0061

Table Supplementary 11: Tukey-multiple comparison results between $\Delta\Delta C_T$ for ALP.

ANOVA table	SS	DF	MS	F (DFn, DFd)	P value
Interaction	13.19	2	6.595	F (2, 12) = 71.24	P<0.0001
Presence/Source of GW	0.4836	2	0.2418	F (2, 12) = 2.612	P=0.1144
Perfusion	0.03125	1	0.03125	F (1, 12) = 0.3375	P=0.5720
Residual	1.111	12	0.09258		

Table Supplementary 12: Two-way ANOVA table results for the BMP2 $\Delta\Delta C_T$ values.

Tukey's multiple comparisons test	Mean 1	Mean 2	Mean Diff.	95.00% CI of diff.	Adjusted P Value
DMSO:Static vs. DMSO:Perfused	-5.330	-7.580	2.250	1.416 to 3.084	<0.0001
DMSO:Static vs. pSi:Static	-5.330	-7.170	1.840	1.006 to 2.674	<0.0001
DMSO:Static vs. pSi:Perfused	-5.330	-6.480	1.150	0.3155 to 1.984	0.0059
DMSO:Static vs. GW:Static	-5.330	-7.410	2.080	1.246 to 2.914	<0.0001
DMSO:Static vs. GW:Perfused	-5.330	-5.600	0.2700	-0.5645 to 1.104	0.8776
DMSO:Perfused vs. pSi:Static	-7.580	-7.170	-0.4100	-1.244 to 0.4245	0.5845
DMSO:Perfused vs. pSi:Perfused	-7.580	-6.480	-1.100	-1.934 to -0.2655	0.0083
DMSO:Perfused vs. GW:Static	-7.580	-7.410	-0.1700	-1.004 to 0.6645	0.9804
DMSO:Perfused vs. GW:Perfused	-7.580	-5.600	-1.980	-2.814 to -1.146	<0.0001
pSi:Static vs. pSi:Perfused	-7.170	-6.480	-0.6900	-1.524 to 0.1445	0.1295
pSi:Static vs. GW:Static	-7.170	-7.410	0.2400	-0.5945 to 1.074	0.9202
pSi:Static vs. GW:Perfused	-7.170	-5.600	-1.570	-2.404 to -0.7355	0.0004
pSi:Perfused vs. GW:Static	-6.480	-7.410	0.9300	0.09551 to 1.764	0.0262
pSi:Perfused vs. GW:Perfused	-6.480	-5.600	-0.8800	-1.714 to -0.04551	0.0367
GW:Static vs. GW:Perfused	-7.410	-5.600	-1.810	-2.644 to -0.9755	0.0001

Table Supplementary 13: Tukey-multiple comparison results between $\Delta\Delta C_T$ for BMP2.

ANOVA table	SS	DF	MS	F (DFn, DFd)	P value
Interaction	0.08392	2	0.04196	F (2, 12) = 0.3769	P=0.6938
Presence/Source of GW	3.637	2	1.818	F (2, 12) = 16.33	P=0.0004
Perfusion	4.107	1	4.107	F (1, 12) = 36.89	P<0.0001
Residual	1.336	12	0.1113		

Table Supplementary 14: Two-way ANOVA table results for the OPN $\Delta\Delta C_T$ values.

Tukey's multiple comparisons test	Mean 1	Mean 2	Mean Diff.	95.00% CI of diff.	Adjusted P Value
DMSO:Static vs. DMSO:Perfused	-2.003	-2.801	0.7980	-0.1170 to 1.713	0.1016
DMSO:Static vs. pSi:Static	-2.003	-2.682	0.6790	-0.2360 to 1.594	0.2007
DMSO:Static vs. pSi:Perfused	-2.003	-3.619	1.616	0.7010 to 2.531	0.0008
DMSO:Static vs. GW:Static	-2.003	-2.910	0.9070	-0.008046 to 1.822	0.0525
DMSO:Static vs. GW:Perfused	-2.003	-4.041	2.038	1.123 to 2.953	<0.0001
DMSO:Perfused vs. pSi:Static	-2.801	-2.682	-0.1190	-1.034 to 0.7960	0.9975
DMSO:Perfused vs. pSi:Perfused	-2.801	-3.619	0.8180	-0.09705 to 1.733	0.0902
DMSO:Perfused vs. GW:Static	-2.801	-2.910	0.1090	-0.8060 to 1.024	0.9983
DMSO:Perfused vs. GW:Perfused	-2.801	-4.041	1.240	0.3250 to 2.155	0.0067
pSi:Static vs. pSi:Perfused	-2.682	-3.619	0.9370	0.02195 to 1.852	0.0437
pSi:Static vs. GW:Static	-2.682	-2.910	0.2280	-0.6870 to 1.143	0.9545
pSi:Static vs. GW:Perfused	-2.682	-4.041	1.359	0.4440 to 2.274	0.0033
pSi:Perfused vs. GW:Static	-3.619	-2.910	-0.7090	-1.624 to 0.2060	0.1700
pSi:Perfused vs. GW:Perfused	-3.619	-4.041	0.4220	-0.4930 to 1.337	0.6427
GW:Static vs. GW:Perfused	-2.910	-4.041	1.131	0.2160 to 2.046	0.0131

Table Supplementary 15: Tukey-multiple comparison results between $\Delta\Delta C_T$ for OPN.

ANOVA table	SS	DF	MS	F (DFn, DFd)	P value
Interaction	7.223	2	3.612	F (2, 12) = 49.19	P<0.0001
Presence/Source of GW	12.42	2	6.210	F (2, 12) = 84.58	P<0.0001
Perfusion	2.431	1	2.431	F (1, 12) = 33.11	P<0.0001
Residual	0.8811	12	0.07342		

Table Supplementary 16: Two-way ANOVA table results for the OCN $\Delta\Delta C_T$ values.

Tukey's multiple comparisons test	Mean 1	Mean 2	Mean Diff.	95.00% CI of diff.	Adjusted P Value
DMSO:Static vs. DMSO:Perfused	-1.434	-2.950	1.516	0.7729 to 2.259	0.0002
DMSO:Static vs. pSi:Static	-1.434	-2.217	0.7830	0.03987 to 1.526	0.0369
DMSO:Static vs. pSi:Perfused	-1.434	-3.958	2.524	1.781 to 3.267	<0.0001
DMSO:Static vs. GW:Static	-1.434	-4.748	3.314	2.571 to 4.057	<0.0001
DMSO:Static vs. GW:Perfused	-1.434	-3.696	2.262	1.519 to 3.005	<0.0001
DMSO:Perfused vs. pSi:Static	-2.950	-2.217	-0.7330	-1.476 to 0.01013	0.0540
DMSO:Perfused vs. pSi:Perfused	-2.950	-3.958	1.008	0.2649 to 1.751	0.0067
DMSO:Perfused vs. GW:Static	-2.950	-4.748	1.798	1.055 to 2.541	<0.0001
DMSO:Perfused vs. GW:Perfused	-2.950	-3.696	0.7460	0.002870 to 1.489	0.0489
pSi:Static vs. pSi:Perfused	-2.217	-3.958	1.741	0.9979 to 2.484	<0.0001
pSi:Static vs. GW:Static	-2.217	-4.748	2.531	1.788 to 3.274	<0.0001
pSi:Static vs. GW:Perfused	-2.217	-3.696	1.479	0.7359 to 2.222	0.0003
pSi:Perfused vs. GW:Static	-3.958	-4.748	0.7900	0.04687 to 1.533	0.0350
pSi:Perfused vs. GW:Perfused	-3.958	-3.696	-0.2620	-1.005 to 0.4811	0.8361
GW:Static vs. GW:Perfused	-4.748	-3.696	-1.052	-1.795 to -0.3089	0.0048

Table Supplementary 17: Tukey-multiple comparison results between $\Delta\Delta C_T$ for OCN.

Scaffold/Culture Condition	DF	Variance	χ^2	P Value
DMSO/Static	5	2.41E-04	5.59439	0.3477
pSi/Static		1.52E-04		
GW/Static		3.33E-04		
DMSO/Perfused		1.46E-04		
pSi/Perfused		5.72E-05		
GW/Perfused		5.38E-06		

Table Supplementary 18: Bartlett's test for homogeneity of variances of bone mineral density (g/cm^3).

ANOVA table	SS	DF	MS	F (DFn, DFd)	P value
Interaction	7.847e-005	2	3.924e-005	F (2, 12) = 0.3776	P=0.6934
Presence/Source of GW	0.01246	2	0.006232	F (2, 12) = 59.98	P<0.0001
Perfusion	0.004984	1	0.004984	F (1, 12) = 47.96	P<0.0001
Residual	0.001247	12	0.0001039		

Table Supplementary 19: Two-way ANOVA table results for the bone mineral density (BMD) (g/cm^3) values.

Tukey's multiple comparisons test	Mean 1	Mean 2	Mean Diff.	95.00% CI of diff.	Adjusted P Value
DMSO:Static vs. DMSO:Perfused	0.03501	0.07331	-0.03831	-0.06626 to -0.01035	0.0062
DMSO:Static vs. pSi:Static	0.03501	0.07719	-0.04219	-0.07014 to -0.01423	0.0029
DMSO:Static vs. pSi:Perfused	0.03501	0.1053	-0.07027	-0.09823 to -0.04232	<0.0001
DMSO:Static vs. GW:Static	0.03501	0.1016	-0.06663	-0.09459 to -0.03868	<0.0001
DMSO:Static vs. GW:Perfused	0.03501	0.1351	-0.1001	-0.1280 to -0.07212	<0.0001
DMSO:Perfused vs. pSi:Static	0.07331	0.07719	-0.003880	-0.03184 to 0.02408	0.9965
DMSO:Perfused vs. pSi:Perfused	0.07331	0.1053	-0.03196	-0.05992 to -0.004008	0.0222
DMSO:Perfused vs. GW:Static	0.07331	0.1016	-0.02832	-0.05628 to -0.0003680	0.0464
DMSO:Perfused vs. GW:Perfused	0.07331	0.1351	-0.06177	-0.08972 to -0.03381	<0.0001
pSi:Static vs. pSi:Perfused	0.07719	0.1053	-0.02808	-0.05604 to -0.0001280	0.0487
pSi:Static vs. GW:Static	0.07719	0.1016	-0.02444	-0.05240 to 0.003512	0.1003
pSi:Static vs. GW:Perfused	0.07719	0.1351	-0.05789	-0.08584 to -0.02993	0.0002
pSi:Perfused vs. GW:Static	0.1053	0.1016	0.003640	-0.02432 to 0.03160	0.9974
pSi:Perfused vs. GW:Perfused	0.1053	0.1351	-0.02980	-0.05776 to -0.001848	0.0344
GW:Static vs. GW:Perfused	0.1016	0.1351	-0.03344	-0.06140 to -0.005488	0.0164

Table Supplementary 20: Tukey-multiple comparison results for BMD (g/cm^3) values.

Scaffold/Culture Condition	DF	Variance	χ^2	P Value
DMSO/Static	5	7.70E+03	14.704	0.0117
pSi/Static		5.38E+04		
GW/Static		2.16E+05		
DMSO/Perfused		1.63E+04		
pSi/Perfused		9.61E+05		
GW/Perfused		1.19E+04		

Table Supplementary 21: Bartlett's test for homogeneity for variances of compressive modulus (KPa).

ANOVA table	SS	DF	MS	F (DFn, DFd)	P value
Interaction	319707	2	159853	F (2, 12) = 0.7578	P=0.4899
Presence/Source of GW	2631396	2	1315698	F (2, 12) = 6.237	P=0.0139
Perfusion	4896900	1	4896900	F (1, 12) = 23.21	P=0.0004
Residual	2531261	12	210938		

Table Supplementary 22: Two-way ANOVA table results for the compressive modulus (KPa).

Tukey's multiple comparisons test	Mean 1	Mean 2	Mean Diff.	95.00% CI of diff.	Adjusted P Value
DMSO:Static vs. DMSO:Perfused	779.2	1667	-887.7	-2147 to 371.9	0.2412
DMSO:Static vs. pSi:Static	779.2	1387	-607.9	-1868 to 651.7	0.6013
DMSO:Static vs. pSi:Perfused	779.2	2805	-2026	-3286 to -766.6	0.0017
DMSO:Static vs. GW:Static	779.2	1541	-761.8	-2021 to 497.8	0.3802
DMSO:Static vs. GW:Perfused	779.2	2365	-1585	-2845 to -325.8	0.0116
DMSO:Perfused vs. pSi:Static	1667	1387	279.7	-979.9 to 1539	0.9717
DMSO:Perfused vs. pSi:Perfused	1667	2805	-1139	-2398 to 121.0	0.0854
DMSO:Perfused vs. GW:Static	1667	1541	125.8	-1134 to 1385	0.9993
DMSO:Perfused vs. GW:Perfused	1667	2365	-697.7	-1957 to 561.9	0.4672
pSi:Static vs. pSi:Perfused	1387	2805	-1418	-2678 to -158.7	0.0245
pSi:Static vs. GW:Static	1387	1541	-153.9	-1414 to 1106	0.9981
pSi:Static vs. GW:Perfused	1387	2365	-977.5	-2237 to 282.1	0.1689
pSi:Perfused vs. GW:Static	2805	1541	1264	4.765 to 2524	0.0489
pSi:Perfused vs. GW:Perfused	2805	2365	440.8	-818.8 to 1700	0.8401
GW:Static vs. GW:Perfused	1541	2365	-823.6	-2083 to 436.0	0.3064

Table Supplementary 23: Tukey-multiple comparison results for compressive modulus (KPa).

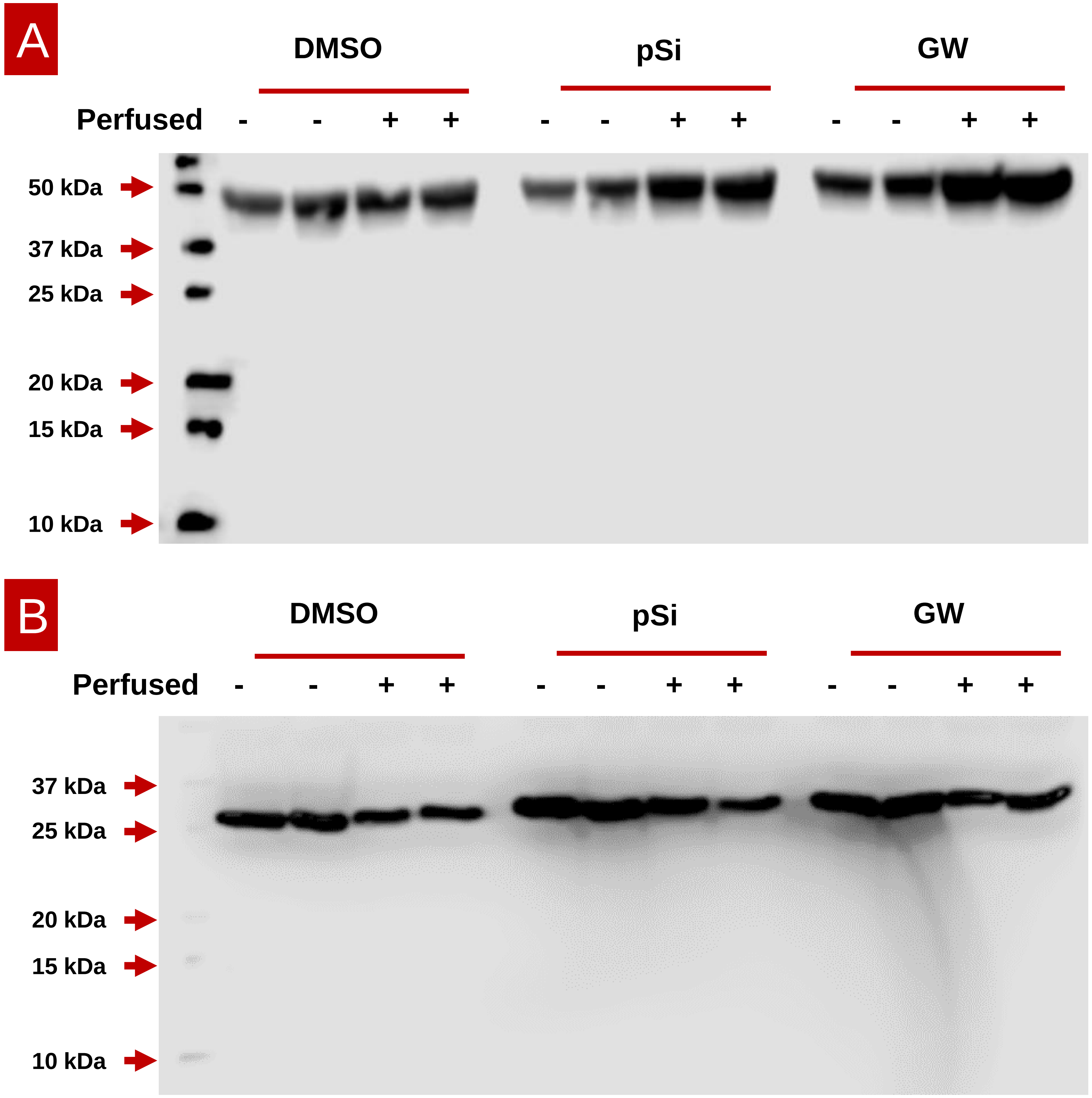

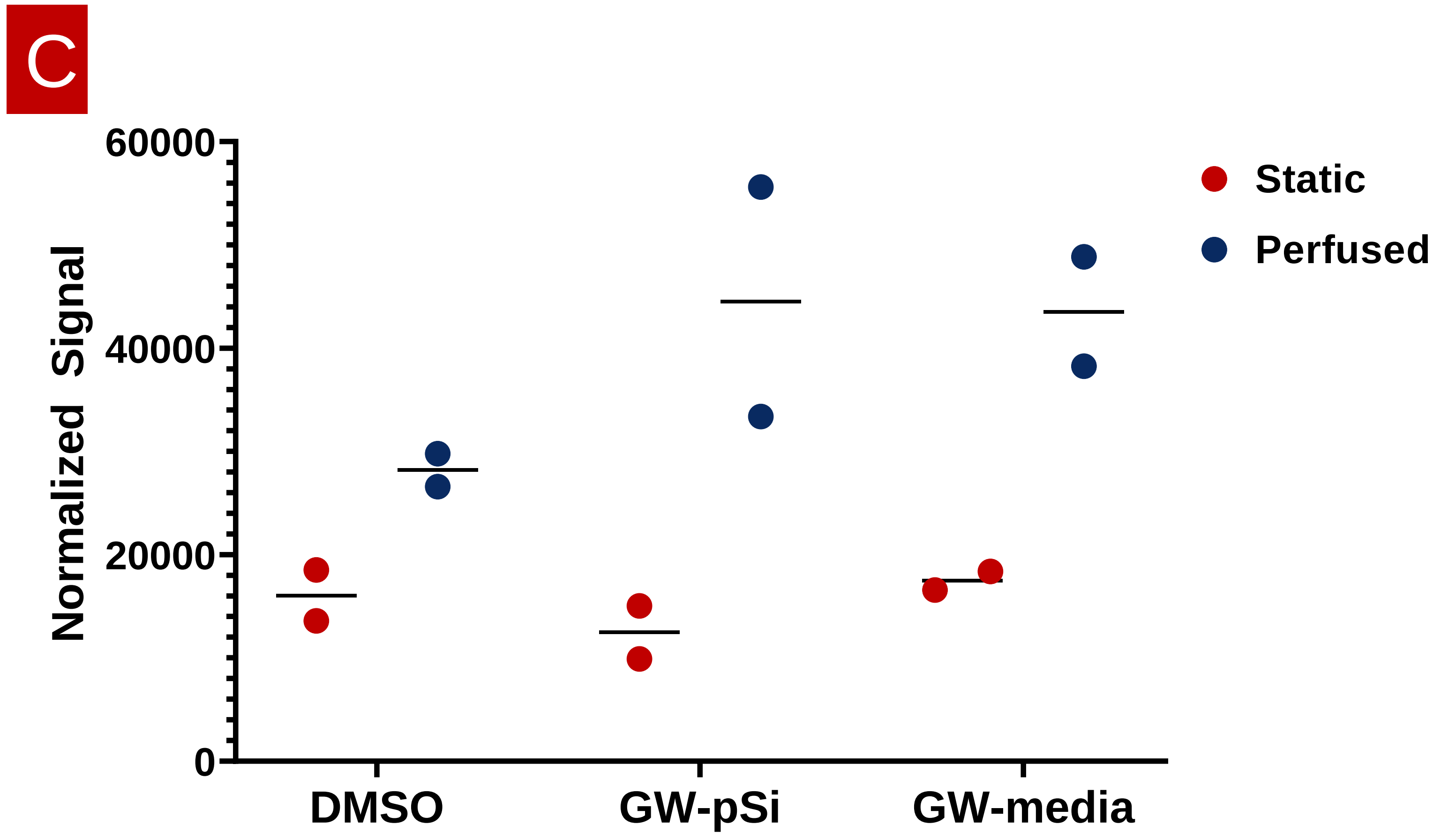

Figure Supplementary 2: Immunoblotting of extracted proteins from various hMSCs/scaffolds combinations after 8 days of culture for A) BMP2 and B) GAPDH. C) Densitometric measurement of BMP2 signal normalized to corresponding GAPDH signal.
